# The Factors Associated With Confidence in Using the Internet to Access Health Information: Cross-sectional Data Analysis

**DOI:** 10.2196/39891

**Published:** 2023-04-11

**Authors:** Kasi Lou Van Heel, Anna Nelson, Daniel Handysides, Huma Shah

**Affiliations:** 1 Dr Kiran C Patel College of Osteopathic Medicine Nova Southeastern University Fort Lauderdale, FL United States; 2 Loma Linda University Loma Linda, CA United States

**Keywords:** confidence, health information access, health information seeking, health information sources, internet, health information

## Abstract

**Background:**

Confidence in health information access is a measure of the perceived ability to obtain health information. One’s beliefs or perceived ability to access health information is particularly important in understanding trends in health care access. Previous literature has found that access to health information is lowest among society’s most vulnerable population groups. These groups include older, less educated, and low-income populations. While health confidence has previously been used as a scale to measure health outcomes, additional research is needed describing the demographic factors associated with users’ confidence in health information access. This may be a key component of health information seeking that affects beneficial health outcomes such as prevention and treatment.

**Objective:**

This study examines the demographic factors associated with the levels of confidence in using the internet to access health information for adults 18 years and older in the United States.

**Methods:**

Using a cross-sectional design, secondary data from the Health Information National Trends Survey (HINTS) 5, Cycle 3 (2019) were analyzed (N=5374). An ordinal regression stratified by internet use was used to determine the association between demographic characteristics and level of confidence in health information access.

**Results:**

When the internet is the primary source for health information, high school graduates (adjusted odds ratio [AOR] 0.58, 95% CI 0.37-0.89) compared to those with a college degree or more had significantly lower odds of being confident in obtaining health information. In addition, non-Hispanic Asian participants (AOR 0.44, 95% CI 0.24-0.82) compared to non-Hispanic White participants, male participants (AOR 0.72, 95% CI 0.54-0.97) compared to female participants, and those who made between US $20,000-$35,000 annually (AOR 0.55, 95% CI 0.31-0.98) compared to those who made US $75,000 or more annually had significantly lower odds of being confident in obtaining health information via the internet. Moreover, when the internet is the primary source for health information, those with health insurance had significantly higher odds of being confident in obtaining health information (AOR 2.91, 95% CI 1.58-5.34) compared to those who do not have health insurance. Lastly, a significant association was observed between confidence in health information access and primary health information source and frequency of visiting a health care provider.

**Conclusions:**

Confidence in accessing health information can differ by individual demographics. Accessing health-related information from the internet has become increasingly more common and can provide insight into health information-seeking behaviors. Further exploration of these factors can inform the science of health education by providing deeper insight into improving access to health information for vulnerable populations.

## Introduction

Existing studies suggest that there is an association between health confidence and health outcomes; patients with higher health confidence feel better, manage their conditions better, and save money on their treatment of care [1]. In this context, however, health confidence is an easy-to-measure proxy for patient engagement; it does not describe the health information-seeking experience. Health information access refers to one’s ability to retrieve health information when needed. Successful engagement in health information seeking requires health information access. Since health-seeking experiences can affect one’s ability to access health information, exploring confidence in health information access may provide insight into improving health outcomes.

Studies suggest measurable benefits emerging from the adoption of health information technology [2,3]. These measurable benefits include the delivery of health information in a practical and potentially low-cost way, allowing consumers to save time and money when looking for health information. While some critics suggest the risks of using information technology for health resources, others highlight the benefits of emerging health information technology [4]. For example, obtaining health information online is highly correlated with visiting a doctor or health care provider and plays a role in decisive medical decisions [5,6]. Searching for health information on the internet has even shown positive effects on an individual’s demand for health care.

Perceived ability to access health information is particularly important in understanding trends in health outcomes. Previous studies have documented differences that exist between groups of health information seekers [7]. One of the most notable is demographics including age, racial and ethnic groups, socioeconomic status, and health coverage status [7]. Recent research found that older adults tend to experience barriers when attempting to access the internet and obtain health-related information, which decreases their intention to use [7]. The authors concluded that older adults tend to experience higher levels of stress, a lack of technical literacy, a lack of desire, and perceived financial cost [7].

As technology continues to develop, new and improved tools make earlier methods for retrieving health information obsolete, less preferred, or considered dated by younger groups [8]. The preference differences between older and younger groups are likely due to generational differences in how technology is used and relied on. Regardless of the cause for preference, the level of trust in preferred sources of health information leaves reason to believe that one’s perceived ability to access health information, or confidence in health information access, may also vary by demographics.

The literature has also revealed differences in health information–seeking behaviors between race and ethnicity groups. For example, African American respondents compared to White respondents were no different in seeking health information and using it when they talked with their doctors [9]. However, a comparison of Latino respondents and White respondents revealed that Latino respondents were significantly less likely to seek health information in general and even less likely to use it after they had talked with their doctors [9]. In terms of gender, primary care consultation rates demonstrated that men were 32% less likely to engage in health information–seeking behaviors than their female counterparts and were higher in groups from more deprived areas, illustrating cost-related differences [10-13].

In a survey distributed to over 60,000 households, it was determined that the costs of seeking health information affect the extent to which consumers seek health information [14]. Uninsured individuals are less likely than insured individuals to obtain information from health care professionals when experiencing a health issue, they may look to sources such as the internet to increase their access to health information [14]. These studies support the idea that the uninsured rely on low-cost sources for health information to avoid high costs or health care debt.

While low cost and convenience describe the benefits of health information technology, these same factors highlight the issue of limited access to health care resources and professional care. Choosing the internet for health information because of cost could ultimately affect one’s confidence in their health information access. Although research has provided compelling evidence of the relationship between various demographics and information access, studies that have examined these patterns prior to the era of advanced technology have failed to consider confidence levels, specifically in terms of one’s ability to access health information. Because of this, it may be important to explore whether confidence in accessing health information is associated with the manner and frequency that people seek health information. Investigating whether access to preferred sources for information is associated with differences in demographics may also lend insight into increasing access to those in need and improving health outcomes.

Many of the findings of previous research suggest an association between demographics and health-seeking behaviors [6]. However, while such evidence exists, studies still fail to explore intersecting relationships between confidence levels of accessing health information, demographic factors, preferred health information sources (eg, health professional or doctor, social media or internet, television, or magazine), and frequency in visiting a provider. The purpose of this quantitative cross-sectional study is to identify the demographic factors associated with the levels of confidence in health information access via the internet for adults in the United States.

## Methods

### Overview

This study was guided by the comprehensive model of information seeking (CMIS) [15]. This theoretical model was designed to predict information-seeking actions such as visiting a health care professional or provider. The CMIS model consists of three major schemas, which include (1) antecedents, (2) information carrier factors, and (3) information-seeking actions [15]. In alignment with the purpose of this study, each study variable matches a component of the model. In the original model, antecedents include demographics, personal experience, salience, and beliefs; however, only demographics and beliefs were explored during this study.

Beliefs represent one’s sense of control or efficacy (ie, confidence in accessing health information) for beneficial health outcomes such as prevention and treatment (ie, visiting a professional care provider). Information carrier factors are the second main components of the CMIS model that are composed of both characteristics and utilities. Each antecedent influences a source’s utility. Utility relates the characteristics of a medium directly to the needs of an individual. In general, utility is important for health information seeking.

### Study Design

This was a cross-sectional study design using secondary data from the Health Information National Trends Survey (HINTS) 5, Cycle 3 (2019). The HINTS is a collection of data regarding citizens’ knowledge of and attitudes toward the use of health-related information.

### Ethical Considerations

Because this study uses a national public data set, informed consent from participants was not required. The data used in this analysis did not include individually identifiable information, and the methods did not involve human participants. Additionally, although the research is not considered regulated research, this study was submitted to an institutional review board for determination.

### Participants

When collecting the original data, HINTS, a self-administered questionnaire, was mailed to individuals throughout the United States. The sampling design consisted of two stages. In the first stage, a stratified sample of addresses was selected from a file of residential addresses throughout the United States using the next birthday method. In the second stage, one adult was selected within each sampled household. The sampling frame consisted of a database of addresses used by Marketing Systems Group to provide random samples of addresses throughout the country. For HINTS 5, Cycle 3, however, an additional push-to-web pilot test was also conducted alongside the fielding of Cycle 3, which was referred to as the web pilot. Of the 5590 questionnaires received, 65 were returned blank, 45 were determined to be incompletely filled out, and 42 were identified as duplicates. The remaining 5438 were determined to be eligible; however, the study population was 5374 after removing those who only answered the survey partially.

### Variables, Instrumentation, and Measurement

For this study, demographic characteristics or covariates that were examined included gender (“male” and “female”), race and ethnicity (non-Hispanic White, non-Hispanic Black or African American/Black, Hispanic, non-Hispanic Asian, and non-Hispanic other), age (18-34 years, 35-39 years, 40-44 years, ≥45 years), education (high school or less, post–high school training other than college, some college, college graduate, postgraduate), and health insurance status (yes or no).

Health confidence was measured by a survey question: “Overall, how confident are you that you could get advice or information about health or medical topics if you needed it?” Respondents were able to answer using a 5-point Likert scale, where 1 was “completely confident” and 5 was “not confident at all.”

Health information sources were measured by a survey question: “Imagine that you had a strong need to get information about health or medical topics. Where would you go first?” When measuring information sources in the survey, respondents were able to select from 12 options (eg, health professional or doctor, social media or internet, television, or magazine).

For this study, this question was used to create a new variable called “Accessing health information via the internet.” Accessing health information via the internet was the dichotomous dependent variable. All respondents who selected “internet” were assigned to one group, and all other responses were assigned to a second group.

The frequency of visiting a health care provider was also a dependent variable measured by the number of doctor visits that participants reported within the past 12 months (none, 1 time, 2 times, 3 times, 4 times, 9 times, or 10 or more times). Records where participants answered “don’t know” or those that contained missing data or multiple responses that were selected in error were excluded to minimize underestimation.

### Statistical Analysis

Weighted frequencies and percentages were calculated for the overall study population. Then, bivariate relations, using chi-square analyses, were examined between primary health information source and the frequency of visiting a provider with confidence in seeking health information. For all analyses, statistical significance was assessed at a *P* value of ≤.05.

An ordinal logistic regression stratifying for the use of the internet to access health information was used to determine the association between demographic characteristics and the level of confidence in health information access for adults 18 years or older in the United States. Odds ratios of one’s level of confidence in health information access using the internet were estimated along with 95% CIs. The model controlled for age, race and ethnicity, gender, education, annual household income, and insurance status. The ordinal logistic regression only included participants who selected the internet as their primary source of health information (n=2663). All analyses for this study were conducted using SAS Version 9.4 (SAS Institute). All analyses also accounted for the complex sampling design of the HINTS 5, Cycle 3 (2019) and were weighted to provide national estimates.

## Results

Table 1 shows frequencies, percentages, weighted frequencies, and weighted percentages of the demographic characteristics among the study population. Overall, 51.38% (3775/7349) of respondents were female, while the remaining 49.62% were male. Most of the respondents had some college education (2949/7305, 40.38%), followed by college graduates or a higher degree (2161/7305, 29.58%) and high school graduates (1703/7305, 23.32%). Next, 63.35% (4339/6851) of the participants were non-Hispanic White, and 31.12% (2311/7428) were aged 50-64 years. In relation to the health insurance status of the respondents, most respondents had health insurance (6838/7466, 91.58%). Lastly, 40.08% (2702/6740) of respondents reported an annual household income range of US $75,000 or more.

Table 2 shows the frequencies and weighted percentages of primary health information source and frequency of visiting a health care provider by confidence in health information access. There was a statistically significant association between primary health information source and confidence in health information access (*P*<.001). Of those who consulted with the internet first when needing information about health or medical topics, 94.56% (3597/3804) were confident and 5.44% (2070/3804) were not very confident. There was also a statistically significant association between the frequency of visiting a health care provider and confidence in health information access (*P*<.001). When examining the frequency of visiting a health care provider, of those that did not frequently visit a health care provider (0 times), 86.53% (1011/1169) were confident in health information access and 13.47% (1575/1169) were not confident in obtaining health information.

Table 3 shows the results of the ordinal logistic regression model stratified by using the internet to obtain health information and controlling for age, race and ethnicity, gender, education, annual household income, and insurance. Predictor variables were tested as a priori to ensure there was no violation of the assumption of no multicollinearity. When the internet is the primary source for health information, high school graduates had significantly lower odds of being confident in obtaining health information when compared to those who were college graduates or more (adjusted odds ratio [AOR] 0.58, 95% CI 0.37-0.89), controlling for age, race and ethnicity, gender, income, and insurance status. Non-Hispanic Asians also had significantly lower odds of being confident in obtaining health information from the internet compared to those who identified as non-Hispanic White (AOR 0.44, 95% CI 0.24-0.82), controlling for age, gender, income, and insurance status. Men had significantly lower odds of being confident in obtaining health information compared to women (AOR 0.72, 95% CI 0.54-0.97), controlling for the same variables. Compared to those who made US $75,000 or more annually, those who made between US $20,000-$35,000 annually had significantly lower odds of being confident in obtaining health information (AOR 0.55, 95% CI 0.31-0.98), controlling for age, race and ethnicity, gender, education, and insurance status. Lastly, when the internet is the primary source for health information, those with health insurance had significantly higher odds of being confident in obtaining health information compared to those who did not have health insurance (AOR 2.91, 95% CI 1.58-5.34), controlling for similar variables.

**Table 1:** Overall study population demographics (N=5374)^a^.

Demographic characteristics		Respondents, n (%)		Weighted, n (%)
**Gender**				
	Male	2208 (42.09)	357,344,215 (48.62)	
	Female	3037 (57.90)	377,588,736 (51.38)	
**Education qualification**				
	Less than high school	328 (6.28)	49,083,771 (6.72)	
	High school graduate	936 (17.93)	170,389,100 (23.32)	
	Some college	1572 (30.12)	294,985,542 (40.38)	
	College graduate or more	2382 (45.64)	216,139,139 (29.58)	
**Race/ethnicity**				
	Non-Hispanic White	3014 (62.87)	433,977,270 (63.34)	
	Non-Hispanic Black	670 (13.97)	77,090,009 (11.25)	
	Hispanic	726 (15.14)	115,992,362 (16.93)	
	Non-Hispanic Asian	220 (4.58)	36,697,852 (5.36)	
	Non-Hispanic others	164 (3.42)	21,342,613 (3.12)	
**Health insurance**				
	Yes	4994 (94.57)	683,812,704 (91.58)	
	No	287 (5.43)	62,841,918 (8.42)	
**Age group (years)**				
	18-34	684 (13.09)	180,877,655 (24.35)	
	35-49	953 (18.25)	181,194,151 (24.39)	
	50-64	1649 (31.57)	231,165,038 (31.12)	
	65-74	1154 (22.09)	87,806,802 (11.82)	
	≥75	783 (14.99)	61,784,740 (8.32)	
**Annual household income (US $)**				
	<20,000	894 (18.84)	123,869,690 (18.38)	
	20,000-34,999	608 (12.82)	76,539,847 (11.36)	
	35,000-49,999	622 (13.11)	91,838,949 (13.63)	
	50,000-74,999	841 (17.72)	111,559,395 (16.55)	
	≥75,000	1779 (37.50)	270,214,860 (40.08)	

^a^Frequencies may be missing due to missing values.

**Table 2:** Frequencies and weighted percentages of primary health information source and visiting a health care provider by confidence in health information access.

		Confident, n (%)			Not confident, n (%)			*P* value		
		Respondents	Weighted	Respondents		Weighted				
**Primary health information source**									<.001^a^	
	Books	96 (90.57)	14,576,983 (95.89)	10 (9.43)		625,432 (4.11)				
	Brochures, pamphlets, etc	92 (92.93)	9,901,126 (96.87)	7 (7.07)		319,463 (3.13)				
	Cancer organization	14 (100.0)	1,094,790 (100.0)	0 (0.00)		0 (0.00)				
	Family	92 (90.19)	12,126,614 (89.24)	10 (9.80)		1,461,993 (10.76)				
	Friend/coworker	39 (82.98)	6,246,732 (85.40)	8 (17.02)		1,067,889 (14.59)				
	Doctor or health care provider	618 (94.93)	82,103,306 (95.12)	33 (5.06)		4,213,888 (4.88)				
	Internet	2523 (94.92)	359,783,760 (94.56)	135 (5.07)		20,707,398 (5.44)				
	Library	11 (91.66)	1,478,355 (67.46)	1 (8.33)		713,041 (32.54)				
	Magazines	24 (96.00)	5,011,858 (96.71)	1 (4.00)		170,752 (3.29)				
	Newspapers	12 (85.71)	1,499,591 (82.50)	2 (14.29)		318,033 (17.49)				
	Telephone information No.	15 (83.33)	1,026,173 (71.13)	3 (16.67)		416,440 (28.87)				
	Complementary, alternative, or unconventional practitioner	13 (81.25)	889,405 (81.22)	3 (18.75)		205,675 (18.78)				
**Frequency of visiting a health care provider**									<.001^a^	
	None	544 (85.80)	101,181,811 (86.53)	90 (14.19)		15,754,644 (13.47)				
	1 time	637 (93.12)	95,525,682 (92.79)	47 (6.87)		7,420,903 (7.21)				
	2 times	901 (93.46)	138,798,363 (94.05)	63 (6.54)		8,787,136 (5.95)				
	3 times	746 (93.13)	94,172,679 (92.65)	55 (6.87)		7,475,449 (7.35)				
	4 times	703 (93.73)	88,595,506 (92.93)	47 (6.27)		6,735,222 (7.07)				
	5-9 times	790 (94.72)	101,011,370 (95.10)	44 (5.28)		5,199,714 (4.89)				
	≥10 times	485 (93.63)	62,461,542 (94.38)	33 (6.37)		3,718,797 (5.62)				

^a^The measure of association is significant at a *P* value of ≤.05.

**Table 3:** Stratified ordinal logistic regression results for confidence in health information access using the internet.

Demographic			Adjusted odds ratio (95% CI)
**Age (years)**			
	18-34	Reference	
	35-49	1.14 (0.74-1.76)	
	50-64	1.06 (0.70-1.61)	
	65-74	1.01 (0.63-1.61)	
	≥75	0.84 (0.46-1.53)	
**Race/ethnicity**			
	Non-Hispanic White	Reference	
	Non-Hispanic Black	1.56 (0.85-2.85)	
	Non-Hispanic Asian	0.45 (0.24-0.82)^a^	
	Hispanic	1.06 (0.68-1.67)	
	Non-Hispanic other	0.81 (0.41-1.60)	
**Gender**			
	Female	Reference	
	Male	0.73 (0.54-0.98)^a^	
**Education**			
	College graduate/more	Reference	
	Some college education	0.96 (0.70-1.33)	
	High school graduate	0.58 (0.38-0.90)^a^	
	Less than high school education	0.43 (0.17-1.09)	
**Annual household income (US $)**			
	75,000	Reference	
	50,000-74,999	0.80 (0.56-1.16)	
	35,000-49,999	1.11 (0.67-1.82)	
	20,000-34,999	0.56 (0.31-0.98)^a^	
	<20,000	0.73 (0.44-1.21)	
**Insurance**			
	No health insurance	Reference	
	Health insurance	2.91 (1.58-5.35)^a^	

^a^The measure of association is significant at a *P* value of ≤.05.

## Discussion

### Principal Findings

This research aimed to determine the factors associated with the confidence levels of individuals seeking health information. In the crude bivariate analysis, results showed a significant association between primary health information source and confidence in health information access. A significant association was also shown between the frequency of visiting a health care provider and confidence in health information access.

In the adjusted multivariable analysis, when the internet is the primary source for health information, participants who were non-Hispanic Asian were significantly less likely to feel confident in obtaining health-related information compared to non-Hispanic White participants. Furthermore, compared to participants with a higher education, those with a high school diploma were significantly less likely to feel confident in obtaining health-related information when using the internet. Additionally, participants with a lower annual household income were significantly less likely to feel confident when using the internet as a source of health information. Lastly, having health insurance was associated with higher odds of feeling confident when using the internet as a resource for health information.

The findings within this study align with previous research that shows demographic factors such as education level, race, and insurance as factors that influence health information consultations [6]. However, the previous studies did not specifically explore the association between using the internet as a source to obtain health information and health confidence levels. Education, income, and race being significantly associated with lower confidence when using the internet as a source to obtain health information may be related to lower levels of health literacy and lower access to health resources among vulnerable populations. Future studies should explore this association further. One approach to increasing health literacy and access to health resources among these populations is to develop tailored community interventions for educational support.

The statistically significant association between having health insurance and being more confident in obtaining health-related information through the internet may be due to insured patients having additional guidance from their health care providers. This finding is further supported by earlier research that found a statistically significant association between the two variables [16]. Additionally, the significance between having health insurance and being confident in obtaining health information through the internet could be attributed to employers and insurance agencies influencing the self-care of patients. These results can indicate that further investigation into having health insurance and access to health information sources can result in strategies that would improve the health outcomes of patients [17].

Limited research describes levels of confidence in health information access, which could provide supporting details for health educators. Researchers have highlighted the benefits of using the internet to increase access to health information, such as immediate educational opportunities regarding the causes, treatment, and prevention of specific diseases and illnesses [18,19]. To promote health and prevent disease, disability, and premature death through education-driven behavior change activities, it is important to recognize how confidence in accessing health information can differ by individual demographics, preferences in sources, and frequency of provider visits.

This study specifically addresses health-related information from the internet, which has become a more common source of health information than in the past. The results of this study inform the science of health education as it details an association between confidence levels and accessing health information using the internet by demographics. This study also allows educational institutions and organizations to better understand confidence levels toward accessing health information from various health information sources as well as the frequency of visiting a health care provider. This study contributes to the current body of literature by addressing confidence in health information access and not just health confidence. This study aids in bridging the gap in the literature to provide insight into health education programs per academia.

### Limitations

One limitation of this study is that data from surveys can rely heavily on self-reported responses, resulting in the potential for recall bias. An additional limitation is the use of a cross-sectional design. Because a cross-sectional design focuses on one specific point in time, causal inferences are not possible [20]. Therefore, future studies should further explore how individuals’ health confidence levels change over time in relation to obtaining health-related information. Lastly, due to a secondary data constraint, the results of this study simply show associations and lack explanations and supporting details.

### Recommendations

One recommendation is that future research collects data from a new subset of surveys from smaller subsets of populations in different geographical regions. Because health confidence changes over time based on factors such as rural, urban, and suburban areas, it would behoove future researchers to focus on these areas individually to gain a stronger understanding of specific changes related to their region. A second recommendation is for future researchers to use a qualitative design, which could provide additional insight into participants’ perceptions, beliefs, and lived experiences. Additionally, future researchers could conduct a longitudinal study that can examine confidence over time. This will provide a further understanding of continuous changes and trends that occur among health information seekers and help understand how confidence levels are influenced by these.

### Conclusions

This research explored the intersecting relationships between demographic factors, confidence levels of accessing health information, health information sources, and frequency of visiting a health care provider. It was found that ethnicity, education, income, and health insurance are associated with one’s confidence level. The study also found that there is an association between the primary source of health information and one’s confidence level in obtaining health information. Lastly, we found that there is an association between the frequency of visiting a health care provider and one’s confidence level in obtaining health information.

## Abbreviations

AOR: adjusted odds ratio
CMIS: comprehensive model of information seeking
HINTS: Health Information National Trends Survey
